# Advances in Synthetic Biology and Biosafety Governance

**DOI:** 10.3389/fbioe.2021.598087

**Published:** 2021-04-30

**Authors:** Jing Li, Huimiao Zhao, Lanxin Zheng, Wenlin An

**Affiliations:** ^1^College of Life Science and Technology, Beijing University of Chemical Technology, Beijing, China; ^2^College of Humanities and Law, Beijing University of Chemical Technology, Beijing, China

**Keywords:** synthetic biology, artificial life, biosafety, regulation and legislation, public health emergency response system, pandemic control strategies

## Abstract

Tremendous advances in the field of synthetic biology have been witnessed in multiple areas including life sciences, industrial development, and environmental bio-remediation. However, due to the limitations of human understanding in the code of life, any possible intended or unintended uses of synthetic biology, and other unknown reasons, the development and application of this technology has raised concerns over biosafety, biosecurity, and even cyberbiosecurity that they may expose public health and the environment to unknown hazards. Over the past decades, some countries in Europe, America, and Asia have enacted laws and regulations to control the application of synthetic biology techniques in basic and applied research and this has resulted in some benefits. The outbreak of the COVID-19 caused by novel coronavirus SARS-CoV-2 and various speculations about the origin of this virus have attracted more attention on bio-risk concerns of synthetic biology because of its potential power and uncertainty in the synthesis and engineering of living organisms. Therefore, it is crucial to scrutinize the control measures put in place to ensure appropriate use, promote the development of synthetic biology, and strengthen the governance of pathogen-related research, although the true origin of coronavirus remains hotly debated and unresolved. This article reviews the recent progress made in the field of synthetic biology and combs laws and regulations in governing bio-risk issues. We emphasize the urgent need for legislative and regulatory constraints and oversight to address the biological risks of synthetic biology.

## Introduction

Synthetic biology emerged at the beginning of the 21st century and has demonstrated huge potentials for basic research and application. Meanwhile, many issues related to synthetic biology’s biosafety and biosecurity need to be deliberated. Over the past decades, the United States, the European Union, and some Asian countries have made great efforts to formulate and implement relevant laws, regulations, and other approaches regarding govern synthetic biology research (Rager-Zisman, 2012; Buhk, 2014). The abrupt outbreak of the COVID-19 caused by the novel coronavirus SARS-CoV-2 at the end of 2019 swept around the world. In the past 1 year, 70.7 million people were infected with SARS-COV-2 and 1.58 million people died from COVID-19, imposing huge impact on the international community. Although the precise origin of the novel coronavirus remains unresolved, in terms of the fast development of synthetic biology techniques, the outbreak of COVID-19 has raised a lot of concerns. This is because these techniques allow researchers to quickly reconstruct or engineer/modify viruses based on available viral sequences (Chen et al., 2019). This article reviews the progress of synthetic biology in several fields, discusses the challenges and bio-risk concerns faced by synthetic biology, compares the current strategies in different countries, and offers suggestions to effectively prevent and curb the pandemic like COVID-19.

## Overview of Progress in Synthetic Biology

### Synthetic Biology

Synthetic biology is an emerging interdisciplinary research field that can broadly be described as the design and construction of novel artificial biological pathways, organisms or devices, or the redesign of existing natural biological systems, aiming to address important issues such as energy, materials, health, and environmental issues. With its rapid development, synthetic biology is becoming a leading third biotechnology revolution since the discovery of DNA double helix and the Human Genome Project. In 2014, the US Department of Defense listed it as one of its six major priority disruptive technologies for development in the 21st century (U.S. Department of Defense, 2014). Advances in biotechnology have promoted the rapid development of synthetic biology. Based on the third-generation genome sequencing, bioinformatics, and gene editing technologies, one can carry out a variety of research tasks using synthetic biology.

This can include genetically engineered life forms thus expanding the fields of genetics and genomics from systems biology to synthetic applications. Rewarding progress on solving medical problems, energy and metabolic engineering, environmental restoration, materials science, and plant science has been made by synthetic biology (Anderson et al., 2012; Liu and Stewart, 2015; Wang L. et al., 2018). In recent years, the in-depth development of cell-free systems has been changing the pattern of synthetic biology and eliminating some of the limitations of working with living cells (Tinafar et al., 2019).

The combination of synthetic biology and engineering improves people’s understanding of the mechanism of action of biological components and the regulatory mechanism of the complex network in organisms (Bashor and Collins, 2018; Ozdemir et al., 2018). Tremendous breakthroughs have been witnessed in genetically engineered living organisms (including pathogens), but this increases potential bio-risks including biosafety, biosecurity, and even cyberbiosecurity.

### Potential Bio-Risks of Synthetic Biology

The potential bio-risks of synthetic biology include biosafety, biosecurity, and cyberbiosecurity. Biosafety initially appeared in the field of microbiology as an abbreviation of safety in biological containment. Thus, the original biosafety definition referred to the safety issue of microbial biocontainment. Later, in the field of transgenic biotechnology associated with genetically modified organisms (GMOs), biosafety emerged as an acronym for “safety in biotechnology,” referring to the safety issues associated with releasing GMOs into the open environment. The term “laboratory biosafety” is often used to refer specifically to safety issues related to the biological laboratory protection of pathogens, GMOs, or genetically modified pathogens. In general, biosafety refers to the safety with respect to the effects of biological research on humans and the environment (Merriam-Webster, 2021). Biosafety emphasizes the prevention of unintentional biotechnological and microbial bio-hazards (World Health Organization [WHO], 2018).

Biosecurity refers to taking proactive measures to avoid intentional biohazards, such as theft and misuse of biotechnology and microbiologically hazardous substances, aiming to reduce the risks associated with the misuse of synthetic biology which could cause harm to humans, animals, plants, and environment through the creation, production, and deliberate or accidental release of infectious disease agents or their byproducts (e.g., toxins). It involves disease control (e.g., vaccination management), exotic species, or access to safe and adequate food supply chains (World Health Organization [WHO], 2010).

Cyberbiosecurity has been proposed as an emerging hybridized discipline which encompasses cybersecurity, cyber-physical security, and biosecurity as applied to biological and biomedical-based systems, and was defined as “understanding the vulnerabilities to unwanted surveillance, intrusions, and malicious and harmful activities which can occur within or at the interfaces of comingled life and medical sciences, cyber, cyber-physical, supply chain and infrastructure systems, and developing and instituting measures to prevent, protect against, mitigate, investigate and attribute such threats as it pertains to security, competitiveness and resilience” (Murch et al., 2018). A more comprehensive definition of cyberbiosecurity is given by Peccoud et al. (2018), who consider that any unforeseeable adverse consequences fostered by the cyber-physical interface can be regarded as a kind of cyberbiosecurity, not merely behaviors related to intentional forms of misuse (Mueller, 2021).

### Bottlenecks in Synthetic Biology Development

Synthetic biology has undergone breakthroughs, but it is still in its early stages, and many of the challenging biological engineering and *de novo* synthesis of life forms are far from perfect due to limitations in knowledge and technology. The use of synthetic biology to reconstruct and synthesize life forms from scratch is currently limited to the following techniques including gene editing or synthesis based on known sequences and functions of already existing life forms, screening libraries for biological components, building regulatory modules and systems, and synthesizing life forms. Synthetic biology techniques need to be closely integrated with comprehensive knowledge of systems biology to enable the precise design, construction, and creation of new life forms. Unfortunately, our current understanding of the fine structure and regulatory mechanisms of life forms is very limited, making the task of designing and synthesizing entirely new life forms from scratch almost impossible.

However, with the development of systems biology, precision gene editing technology, bioengineering technology, bioinformatics including multidimensional genomics, big data analysis, and artificial intelligence, the modification and synthesis of life forms might be helpful in breaking through the technical and cognitive bottlenecks currently faced by current synthetic biology. Unfortunately, at present, there is no evidence to prove that one can design and synthesize entirely new life forms, including viruses and other pathogens, without designing them based on known genome sequences and functions. The engineered life forms (including plants, animals, microorganisms, etc.) mentioned in this review are either limited modifications of existing life forms based on known gene sequences or *de novo* designed and chemically synthesized based on known genome sequences.

As mentioned above, it is almost impossible to synthesize an entirely new life form using current synthetic biology techniques. However, it has long been a debated concept over whether a GMO can be considered as a new organism. It is not very convincing to infer the origin of a new virus on the basis that one cannot currently synthesize a lifeform from scratch. Nevertheless, the public (and everyone else) is concerned about the actual biological risks of synthetic biology and whether Dual Use Research of Concern (DURC)/GoF research will lead to evolutionary acceleration and recombination, with unintended/undesirable consequences. The crucial bottleneck in synthetic biology is lacking full knowledge of the code of life and the limitations of technology.

Due to the importance of synthetic biology and general supports and endorsements from governments, breakthroughs in synthetic biology bottlenecks require innovative and comprehensive development of cross-disciplines. Therefore, for bio-risks concerns, it is necessary to enhance the implementation of laws and regulations to regulate synthetic biology researching activities and the applications of synthetic biology.

## Benefits and Potential Concerns of Synthetic Biology to Human Society

### Medical Research

The in-depth intersection of synthetic biology and medical fields has created a variety of diagnostic and therapeutic approaches. It provides a new research direction for timely and treatment of diseases in the future. Engineered cells or bacteria are used for diagnosis and therapeutic purposes (Danino et al., 2015; Xie et al., 2016). The use of Salmonella, which carries genes for synthetic antitumor drugs, to control tumor growth by sensing the tumor hypoxic microenvironment and releasing drugs in a time-dependent manner is an innovative idea for cancer therapy (Din et al., 2016). Engineered phages could be used to kill antibiotic-resistant pathogens (Yehl et al., 2019). Researchers have developed a new type of tumor-attacking virus that not only kills tumor cells in the brain, but also blocks the growth of blood vessels in tumors, suggesting that the tumor-killing oncolytic viruses may be more effective in treating aggressive brain tumors if they carry vasculostatin, a protein that inhibits the growth of blood vessels (Hardcastle et al., 2010). Synthetic circuits could be integrated into host cells and function as bio-sensors and regulators for cellular homeostasis or disease progression manipulation (Bai et al., 2016; Saxena et al., 2016). Application of engineered T cell by synthetic biology techniques, for example, Chimeric Antigen Receptor T-Cell Immunotherapy (CAR-T) could be used to treat leukemia (Fraietta et al., 2018). In addition, the idea of controlling engineered cells through external electronic system has been realized with the combination of synthetic biology and computer science (Shao et al., 2017).

Although synthetic biology has been developing rapidly in the medical field and has achieved several breakthroughs, rushed attempts to perform without adequate consideration of bio-risks issues at synthetic biology have also resulted in unintended deaths of patients, and unintended side effects due to individual differences in the use of synthetic biology in therapy still need to be taken seriously (Morgan et al., 2010).

As a novel gene editor, CRISPR/Cas makes it easier to edit sequence-specific genes, which has enabled the development of genetic models of disease and the study of therapeutic measures for human genetic diseases. However, the application of this gene-editing technology and its potential to cause genetic manipulation of humans and human embryos, CRISPR-modified cells and organisms have drawn focused attention to the implications for human biology and society (DiEuliis and Giordano, 2017). Therefore, the premature clinical applications may have erroneous effects.

In addition, gene-editing tools or synthetic biology could be used to enhance (*in vivo* or *in vitro*) production of traditional or novel neurotoxins or infectious agents or to modify existing agents that are known to act on the nervous system and brain to alter neural phenotypes that influence cognition, emotion, and behavior, which raise biosecurity concerns. Gene editing techniques are categorized as a potential vector or even an element of bioweapons of mass destruction (Servick, 2016), and it may be a potential game-changer for neuroweapons (DiEuliis and Giordano, 2017).

### Metabolism of Engineered Organisms

Synthetic biology has made new progress in the field of metabolism of organisms including bacteria, yeast, and mammalian cells through the integration of artificial metabolic networks into engineered strains. To reduce redundant metabolic pathways for better product conversion rates, some researchers have proposed the deletion of unnecessary genes in cells to build smaller genome organisms. Consequently, these organisms are customized to design and produce the desired products (Sung et al., 2016).

*Escherichia coli* has become an important research object in synthetic biology due to its fast growth rate and high conversion rate. The yield of target products increases dramatically in *E. coli* with engineered metabolic networks (Bogorad et al., 2013; Lin et al., 2015; Mahr et al., 2016). By now, a large number of engineered strains have been developed through the introduction of a metabolic network to produce biochemicals, biomaterials, biomedicine, and bioenergy (Saïda et al., 2006; Atsumi et al., 2008; Steen et al., 2010; Huo et al., 2011; Choi and Lee, 2013; Yao et al., 2013; Studier, 2014; Baeshen et al., 2015; Hadadi et al., 2016; Gileadi, 2017).

As the first eukaryote to complete genome sequencing, *Saccharomyces cerevisiae* is a model organism widely used in industry. With the application of novel gene editing technologies such as CRISPR/Cas, engineered *S. cerevisiae* could be used to produce a variety of chemicals. A representative success case is the manufacturing of artemisinin precursor, artemisinin acid (Ro et al., 2006; Heidari et al., 2017). Moreover, researchers have been able to fully synthesize opioids in yeast (Galanie et al., 2016). The production of some rare medicinal extracts can also be synthesized through basic raw materials in engineered *S. cerevisiae* as a factory (Yan et al., 2014; Wang et al., 2015; Wei et al., 2015), significantly reducing the cost of extracting compounds from medicinal reservoirs. This method can replace large-scale cultivation of medicinal plants, hence, an important application of synthetic biology in the field of compound production (Zhao et al., 2019).

Synthetic biology plays a significant role in the pharmaceutical industry, food additives, cosmetics, and energy industries with renewable production methods, driving the development of human industry. The benefits of advances in genetic engineering technology, however, do not allay concerns about the development of synthetic biology, as the products of genetic engineering have significantly altered the life characteristics of engineered living organisms. In particular, the bio-risk aspects and any unexpected effects have not been fully analyzed in the strains which have been engineered by reducing metabolic pathways or deletion of unnecessary genes because of the uncertain consequence of the deletion of “necessary gene,” which might turn out to be necessary in some unrecognized context (Schumacher et al., 2020).

There are two types of biomolecular-based products being developed, including GMOs (e.g., organisms expressing bio-pesticides) and topical chemical or physical formulations for use in medicine, agriculture, and food production or preservation (e.g., antibiotics and biopesticides). Vectors harboring nucleic acids (DNA and RNA) and proteins that destroy or repair DNA for engineering can be applied to penetrate living cells, tissues, and organisms. Adequate assessment of the potential for these technologies to unintentionally cause harm to human health or the environment may be lacking, or may be reassigned to cause harm. Biologically active substances and vectors may escape risk assessment and regulatory review because they are often excluded from the hazardous chemical category and are explicitly excluded from the category of “genetically modified agents.” This emerging oversight loophole could lead to dual-use allocations or unintended harm to human health or the environment (Heinemann and Walker, 2019).

In addition, their environmental invasion capabilities and evolutionary potential are difficult to determine. Under the pressure of natural evolutionary and natural force, back-mutations and the entire spectrum of unintended effects are also inevitable, leading to loss of function of the engineered organism or unexpected consequences. Furthermore, the escape of engineered strains from the laboratory that produce harmful organic matter might cause ecosystem disruptions, and the escape of strains carrying man-made removable elements might even amplify the resulting cascade of harm, disrupting the ecological balance in unforeseen ways. Besides, inhalation or contact ingestion caused by the application of these substances to insecticides or sprays may cause uncertain hazards, such as off-target effects of active ingredient concentration or gene silencing.

According to an American report, no known harmful effect from eating genetically modified foods (National Academies of Sciences and Medicine, 2016) has been affirmed. While the Russian Federation launched the GMOs of plant origin safety assessment system. This system accumulates all national and international experience and the latest scientific approaches and achievements to provide the most complete and reliable information on the potential genotoxic, immunotoxin, and allergenic effects of GMOs and be able to reveal the possible unintended effects of genetic modification (Tyshko and Sadykova, 2016). However, the long-term effects of genetically modified animal and plant products on human genetics and health are also unknown (Engdahl, 2013).

Recently, Bauer-Panskus et al. (2020) summarized the new challenges that arise in risk assessment when genetically engineered (GE) plants can persist and propagate in the environment as well as produce viable offspring, and pointed out that next generation effects may be influenced by heterogeneous genetic backgrounds that may trigger unexpected effects in interaction with environmental conditions. The biological characteristics of the original event cannot be considered sufficient to conclude the possible hazards of the next generation. Potential hazards identified by the European Food Safety Authority (EFSA) include exacerbation of weed problems, displacement, and even extinction of native plant species, resulting in a reasonable concern that might escape environmental risk assessment (ERA) because EFSA considers only the characteristics of the original events, leaving aside unexpected or unintended next-generation effects emerging from spontaneous propagation and gene flow. Therefore, the risk assessment of GE organisms capable of persistent and spontaneous propagation in the environment is in fact highly spatiotemporally complex, causing many uncertainties (Bauer-Panskus et al., 2020). In addition, biological actives used in technologies that allows DNA, RNA, and proteins to be delivered to cells, tissues, and organisms in the open environment may evade risk assessment and regulatory review because they are often excluded from the category of hazardous chemicals and are actively being excluded as agents of genetic modification. This emerging oversight vulnerability could lead to dual use or unintended harm to human health or the environment (Heinemann and Walker, 2019). As we cannot get an affirmative answer on the question whether all possible genetically modified foods are safe, we could not foresee what it would bring about if artificial life was released in nature.

### Environmental Monitoring and Bio-Remediation

With extensive use of antibiotics and organic pesticides, the treatment of refractory pollutants has attracted great attention. Taking synthetic biology as a technical basis, diverse cell factories have shown great potentials in dealing with these severe environmental pollution issues, which is mainly in environmental monitoring and bio-remediation.

In terms of environmental monitoring, engineered cells are used as pollutant monitors. They are sensitive to specific substrates (pollutants) and convert them into human-recognizable signal outputs so that the degree of environmental pollution can be detected. Engineered *Pseudomonas putida* has been used to monitor the concentration of naphthalene in water and gas phases (Werlen et al., 2004). Through continuous optimization of *E. coli* strains, the detectable concentration of parathion, the main substance of organophosphorus pesticides by the engineered biosensor, has reached 10 μmol/L (Chong and Ching, 2016). Concerning environmental bio-remediation, the engineered bacteria through synthetic biology can attain a complete degradation of organochlorine pesticide hexachlorophenol (Yan et al., 2006). DDT pollutants can also be degraded by engineered bacterial strains to stable 4-chlorobenzoic acid metabolites (Kamanavalli and Ninnekar, 2004).

Most bacteria rapidly reproduce in large numbers, and their ability to evolve and mutate allows them to survive in many artificially imposed or naturally occurring environmental stresses. It is difficult to predict how an engineered strain that escapes from the laboratory or is released into the environment will evolve and mutate under the selective pressures of the external environment. Therefore, the biosafety and biosecurity of engineered bacteria or other organisms modified by synthetic biology is currently a matter of great concern in many countries.

Commonly used methods to control of engineered bacteria include implanting a biosafety module in the engineered bacteria, which can trigger the death of the engineered bacteria or prevent the bacteria from self-replication after an escape (Jia et al., 2017). For engineered bacteria that are truly used in the environment, biological control methods, and efficiency need to be evaluated in a timely manner to avoid irreversible consequences.

### *De novo* Synthesis of Living Organisms

Well-established techniques such as gene sequencing and gene synthesis have promoted the rapid development of synthetic biology. Scientists have begun attempts to synthesize living organisms from scratch instead of solely modifying the genome of a living organism. Recent progress shows that scientists have achieved a leap from the synthesis of prokaryotes to eukaryotes.

On May 20, 2010, Gibson et al. (2010) published a paper in the journal Science on the first artificial synthetic cell named “Synthia,” which is a synthetic filamentous mycoplasma, and this artificial cell exhibited the expected characteristics and was capable of self-replication. On May 16, 2019, Chin’s team (Fredens et al., 2019) synthesized a four-mega base *E. coli* genome, and then transformed it into a bacterium called “Syn61” that uses only 61 codons. The synthetic bacteria exhibited a complete viability. Qin’s team (Shao et al., 2018) connected the 16 natural chromosomes of *S. cerevisiae* one by one and deleted redundant repeats, creating a simple yeast SY14 with only a single chromosome, which is different from normal forms of yeast genome. A complete chemical synthesis of 4 *S. cerevisiae* autosomes was achieved in 2017, establishing a rapid customized synthesis technology for long eukaryotic chromosomes (Wu et al., 2017; Xie et al., 2017).

The application of synthetic biology techniques to synthetic life forms is extremely attractive for people to explore and trace the world of life. We may speculate that in the future, it might be possible to synthesize animal and plant varieties with full consideration of any associated bio-risks, which will greatly facilitate people’s understanding of the mysteries of life. However, at current development stage of synthetic biology without a complete understanding of the possible modes of operation and evolution of life, the impact of synthetic life forms on humans, society, and the environment are largely unknown, especially the potential concerns involving those artificially life forms themselves and other life forms. Therefore, the healthy development of synthetic biology still needs to consider a broader range of biosafety and biosecurity issues.

### Modification and Artificial Synthesis of Pathogens

With the development of synthetic biology, the modification and synthesis of pathogens based on the published pathogen’s gene sequence have become a reality. Cello et al. (2002) used chemical methods to synthesize a full-length poliovirus cDNA, which was transcribed into RNA by ordinary RNA polymerase. They successfully obtained the infectious virus by incubating transcribed RNA from poliovirus cDNA with cytoplasmic extracts of Hela cells. Tumpey et al. (2005) successfully synthesized the Spanish influenza virus. The team integrated all the eight coding gene segments of the virus into the genomic DNA of a common influenza virus based on a publication of the 1918 Spanish influenza virus genome sequence. The virus particles were then obtained from human kidney cells injected with the influenza virus containing eight gene fragments (Tumpey et al., 2005).

To study the mechanism of viruses, Menachery et al. (2015) applied reverse genetics approaches to insert bat coronavirus SHC014 spike protein into mouse adaptive SARS-CoV skeleton, so that the virus skeleton can recognize the ACE2 receptor via SHC014 spike protein and be successfully replicated in human airway cells. Virologist David Evans synthesized horsepox virus (an orthopox virus related to variola, but not variola), which was obtained by inserting the DNA into cells using recombinase (Noyce et al., 2018). Recently, scientists from Switzerland, Germany, and Russia reported the use of public available SARS-CoV-2 sequences to rapidly reconstruct the novel coronavirus in yeast (Thao et al., 2020).

As mentioned above, the current synthetic biology technology has not been able to achieve a complete *de novo* design and synthesis of a brand new virus. Nevertheless, *de novo* synthesis of viruses based on the existing viral genome sequences, or modification of the existing viral genome by splicing, varying toxicity, enhancing immune escape, changing incubation period, and target, as well as creating virus mutation libraries can be achieved within a short period of time. Nevertheless, the synthesis of pathogens based on existing or modified genome sequences alone has its inherent limits as it ignores all the vast other steps and mechanisms life uses for plasticity and diversification.

Although synthesis of pathogens can facilitate scientific research on viral pathogenesis and drug development, modification or synthesis of pathogens is a dangerous endeavor, and any report of virus synthesis is accompanied by a great deal of discussion and controversy about biosafety and biosecurity issues (Couzin, 2002; Sharp, 2005; Thiel, 2018). Biosafety and biosecurity and even cyberbiosecurity during pathogen synthesis are big issues that need to be considered in any case. Avoiding any misuse, abuse, and accidental release of pathogens is of paramount importance in the conduct of pathogen research.

## Challenges and Opportunities for Synthetic Biology

Over the past two decades, synthetic biology has changed radically, due to its reliance on digitization and automation, offering powerful tools to engineer and even synthesize life forms. There have been quite some efforts to highlight the resulting dangers in this aspect. These concerns including biosafety, biosecurity, and cyberbiosecurity can no longer be ignored as this is how synthetic biology has been carried out.

Considering the unprecedented positive contributions of synthetic biology to people’s lives, health and the environment, and the concerns raised by its application, we should take proper measures to embrace the opportunities and challenges presented by synthetic biology. The healthy development of synthetic biology will ultimately depend on resolving actual and perceived concerns regarding its biosafety and biosecurity, as well as the potential consequences of the accidental or deliberate release of synthetic biology-derived organisms.

Firstly, synthetic biology does not always achieve the desired results. Synthetic lifeforms are normally made according to human preferences or purposes, and to some extent, it might be also an accelerated process of natural evolution. However, due to our limits of life-code knowledge, the process and outcome of synthetic lifeforms will not always proceed as we designed. Allowing nature to unfold under the evolutionary accelerated pressure is an experiment where it is not clear exactly what will happen. A typical example is the gene editing event in human babies. It is true that the original intent of the technology was to solve the problem of human genetic diseases, but gene editing of human embryos carried out without proper ethical and biosafety evaluation raises serious biosafety concerns. According to the 2020 report “Heritable Human Genome Editing” from an international commission (Medicine, and Sciences., 2020), and the earlier 2017 report “Human Genome Editing: Science, Ethics, and Governance” (National Academies of Sciences et al., 2017), strict ethical evaluations and steps should be taken before proceeding to clinical human germ-line editing. Family, ethical, moral, and religious dimensions are all factors that should be taken into consideration, in addition to essential factors like scientific and medical safety assessments and regulatory frameworks (Medicine, and Sciences., 2020).

Secondly, the explosive growth of sequence information and the sharing mode of a large amount of genetic information have made the synthesis of pathogens much easier. The accessibility and openness nature of the web offers opportunities to anyone who wants to access a dangerous organism. At the same time, intentional data-information errors in information technology have the potential to cause major security problems in biology. With the increased automation of life sciences, the convergence of new biotechnology and information technology may have even more serious consequences (Dunlap and Pauwels, 2017; George, 2019; Murch and DiEuliis, 2019; Mueller, 2021). In the fourth industrial revolution, the intelligent and its connections to bio-labs open the risks of nefarious use to engineer or edit biological agents or toxins. With the combination of synthetic biology with artificial intelligence and automation, the bio-risks of synthetic biology do not only include intended attacks, but also unintended consequences due to the cyber-overlap and automation. Given advances in DNA synthesis techniques and the advent of robotic cloud laboratories, one may find ways to circumvent current governance barriers to enhance the virulence and transmissibility of pathogens (Dunlap and Pauwels, 2017).

Thirdly, the synthesis of live viruses can help people understand the pathogenic mechanism of pathogens and conduct targeted vaccine and drug development to deal with potential outbreaks. However, the synthetic technology of pathogens could aids bio-terrorism. Furthermore, we cannot guarantee that the pathogen sequences synthesized using pathogen synthesis techniques will be identical to the designed pathogen sequences, as any errors in the synthesis process and in the cyber/physical/natural interface could lead to unexpected mutations and unknown consequences.

A slight modification of the virus genome may result in a mutant virus with increased virus latency, increased pathogenicity, increased number of receptor recognition sites, more serious immune escape, and random mutations creating a mutant virus library, thus a serious consequence. Simplification of synthetic biology techniques creates additional safety risks, but the exposure of the problem can guide the elaboration of legislation and help promote further development of synthetic biology.

Finally, the mutation and gene recombination of pathogens are inevitable. Genome mutations and genetic recombination that occur between different pathogens are active to survive and adapt to the environment. Scientists use synthetic biology techniques to directly mutate the pathogens’ genome to accelerate their evolution for scientific purposes, but sometimes the consequence of pathogen engineering is unpredictable because of the limits of individual knowledge about life code.

No matter whether it is the natural recombination of the pathogen genome, virus engineering, or even artificial synthesis in the laboratory, new pathogenic organisms could be produced. Therefore, biosafety and biosecurity are issues that must be highly evaluated for the healthy development of synthetic biology. Restrictions on pathogens and the parallel development of real-time detection technologies are equally urgent. Synthetic biology’s development and imbalance of restrictions will have more impacts on industry, and more regulations and discussions are necessary. There is an urgent need to curb bio-risks by laws and regulations.

## Biosafety Governance of Synthetic Biology in Different Countries

It is difficult to have precise definitions and legislations over synthetic biology because it is intertwined with various disciplines. Nevertheless, the legislation of synthetic biology in various countries are managed to be issued according to the research and application fields of synthetic biology.

### Status of Laws and Regulations on Synthetic Biology in the United States

The United States government has promulgated policies, regulations, and laws governing different biological products. For example, pathogens are classified based on their virulence levels and the availability of vaccines and/or effective anti-pathogen drugs. Various levels of physical containment have been mandated depending on pathogen classifications. In terms of laboratory managements, the US Centers for Disease Control and Prevention (CDC) and the National Institutes of Health (NIH) have published a manual on recommendations for the physical containment of pathogens entitled “Biosafety in Microbiology and Biomedical Laboratories” (Berns, 2014).

All medicines in the United States are governed by the Federal Food, Drug, and Cosmetic Act (FDCA). Specifically, Chapter 5 of the FDCA outlines the regulatory approval and testing of pharmaceuticals including specifications, labeling, safe handling, and directions for the safe use of such drugs, as well as requirements for clinical trials. Others such as Toxic Substance Control Law, Plant Insect Law, etc., oversee relevant departments (Trump, 2017).

After the anthrax attacks in 2001, the United States has introduced a series of laws and regulations covering many areas such as biosecurity threat prevention, biosafety drug development, and dual-use technology regulation. These laws and regulations include the Public Health Security and Bioterrorism Preparedness Response Act of 2002, the Bioshield Act, the Biological Defense and Pandemic Vaccine and Drug Development Act, the National Bioengineered Food Information Disclosure Standard, the U.S. Government’s Regulatory Policy for Life Sciences Dual-Use Research, and other laws and regulations.

Concerning the supervision of scientific life research, the United States government issued the policy of DURC. Life science is a research that, based on current knowledge, can reasonably be expected to provide knowledge, information, products, or technologies that could be directly misapplied to pose a significant threat, with a wide range of potential consequences, to public health and safety, crops and other plants, animals, the environment, materials, or national security.

There are two United States policies on dual-use research of concern. One is the United States Government Policy for Oversight of Life Sciences DURC (U.S. Department of Health & Human Services, 2015). Being released on March 29, 2012, it established a United States Government policy for DURC as applied to a well-defined subset of life sciences research that involves 15 agents and toxins and seven categories of experiments and established regular review by Federal agencies of United States. The other is United States Government Policy for Institutional Oversight of Life Sciences DURC, which was released by the United States government on September 24, 2014. Institutional oversight of DURC is critical for a comprehensive oversight system. This policy ascertains the responsibility of institutions to oversight life sciences DURC, because institutions are most familiar with the life sciences research conducted in their facilities. Also, they are the most proper agencies to shoulder the responsibilities of promoting and strengthening research and communication in the field of life sciences.

These two DURC Policies are complementary to strengthen review and oversight of life sciences research to identify potential DURC, and to develop and implement risk mitigation where appropriate and as required by federal regulation. It supplements the existing regulations and policies of the United States government on the possession and handling of pathogenic microorganisms, and provides guidance to related individuals, including researchers, national security officials and global health experts. They emphasize a culture of responsibility by reminding all involved parties of the shared duty to uphold the integrity of science and prevent the misuse of synthetic biology.

### Status of Laws and Regulations on Synthetic Biology in the European Countries

According to the current EU legislation on GMOs, most of the research conducted in the field of synthetic biology is genetic engineering. This law regulates how organisms are genetically modified and how GMOs are used, including the marketing of GMOs and their products (Buhk, 2014). To limit the scope of application of the law, the EU has set up a special working group for considering applications of new biotechnology in plant breeding and other biological modifications. The European Union has formulated a series of directives for GMOs and emerging biotechnology covering labels, proper containment, trans-shipment, and safe use in research environments (Keiper and Atanassova, 2020).

The European Union’s legislation on the use and regulation of GMOs is mainly based on Directive 90/219/EC, which regulates the genetic modification activities of microorganisms and their cultivation, storage, transport, destruction and disposal, and Directive 2001/18/EC, which regulates the intentional release of GMOs. Although the EU legislation on synthetic biology has been continuously updated over the last two decades, the EU legal framework has been criticized as not being sufficiently comprehensive in scope, and proposals have been made to modify it to suit the rapid development of biotechnology and the new era of synthetic biology (Eriksson et al., 2018; Bratlie et al., 2019; Eriksson et al., 2020).

### Status of Laws and Regulations on Synthetic Biology in China

Synthetic biology is developing very fast in China, especially in the fields of advanced bio-manufacturing, microbial genome breeding, industrial enzyme engineering, and biomedicine. Considering the bio-risk concerns, China has promulgated laws and regulations on biosafety governance of synthetic biology for laboratory practice to secure biosafety and biosecurity (Table 1). The Standing Committee of the National People’s Congress, the representative of China’s supreme legislature, promulgated the Biosafety Law of the People’s Republic of China in 2020. Chapters 4 and 5 of the Biosafety Law generally stipulate the security management of biotechnology research, development, and application activities, and of pathogenic microbe laboratories and formulate unified laboratory biosecurity standards. Any administrative regulations, local regulations and department rules cannot contravene this Biosafety Law. The Ministry of Science and Technology drafted the Regulations for the Safety Management of Biotechnology Research and Development (Biotechnology Research Regulations) right after the gene-edited baby event caused by a Chinese scientist to promote and guarantee the healthy and orderly development of China’s biotechnology research and development activities and maintain national biosecurity. The gene-editing experiment seriously violated academic ethics and standards. The Procuratorate found that it was in a dilemma that there was no suitable law to convict Jiankui He guilty as no such a law existed to prohibit scientists from conducting gene-editing experiments on humans back then. The draft of the Biotechnology Research Regulations will fill this legal gap. In addition, a series of policies have been issued on the prevention and control of infectious diseases and laboratory management. Accordingly, various studies are supervised and managed per the grade standards; adequate risk assessments should be conducted during the transformation of research into practical applications to avoid major biosafety and biosecurity risks.

**TABLE 1:** List of the regulations on biosafety governance of synthetic biology.

Laws and regulations	Issuing authority	Date of enactment
Biosafety Law of the People’s Republic of China	The Standing Committee of the National People’s Congress	Issued on October 17, 2020.
Regulations on Administration of Agricultural Genetically Modified Organisms Safety	State Council	Issued on May 23, 2001, amended in 2011 and 2017.
Regulations on the Biosafety Management of Pathogenic Microbiological Laboratories	State Council	Issued on November 12, 2004, amended in 2016 and 2018.
Measures for the Administration of the Safety Evaluation of Agricultural Genetically Modified Organisms	Ministry of Agriculture (dissolved, with its authorities having been assumed by Ministry of Agriculture and Rural Affairs)	Issued on January 5, 2002.
Working Rules of the Agricultural Genetically Modified Organisms (GMO) Safety Committee	Ministry of Agriculture (dissolved, with its authorities having been assumed by Ministry of Agriculture and Rural Affairs)	Issued on May 17, 2013.
Guidelines for Veterinary Laboratory Biosafety	Ministry of Agriculture (dissolved, with its authorities having been assumed by Ministry of Agriculture and Rural Affairs)	Issued on October15, 2003.
Measures for the Biosafety Environmental Management of Pathogenic Microbe Laboratories	State Administration of Environmental Protection (dissolved, with its authorities having been assumed by Ministry of Ecology and Environment)	Issued on March 08, 2006.
Measures for the Safety Management of Biotechnology Research and Development	Ministry of Science and Technology	Issued on July 12, 2017.
Biosafety Guidelines on the Biosafety Governance of Novel Coronavirus High-grade Viral Microbiology Laboratory	Ministry of Science and Technology	Issued on January 23, 2020.

### Requirement of International Cooperation in the Prevention and Control of Bio-Risks

As globalization proceeds, the international cooperation regarding biosafety governance is necessary. To respond to the possible risks and threats from synthetic biology, countries are highly recommended to strengthen various measures such as strengthening the top-level design of biosafety, stepping up the formulation and improvement of national laws and policies. In addition, they are urged to focus on the overall biosafety and biosecurity education layout such as talent training and discipline creation to achieve a profound contribution to technology governance around the world.

The administration departments including government regulatory agencies, government funding agencies, and research institutions should improve the review system of scientific research projects, conduct potential risk analysis, and focus on engineering pathogenic microorganisms and biological research that may have a negative impact on the natural environment, as well as research with ethical issues. The development of any project related to pathogens has potential biosafety risk. The corresponding review of the research proposal should be carried out before starting a project, in the process of implementation and during the translation of research results into practical applications. It is necessary to comprehensively evaluate the impact of bio-risks on natural environment and human society and take positive actions to guarantee national security and public interest whilst promoting the development of synthetic biology.

The Center for Biological Safety Strategic Research of Tianjin University in China and the Johns Hopkins Center for Health and Safety of the United States co-sponsored the “Track II dialogue” entitled “The Challenges Facing China and the United States in the Era of Synthetic Biology” in 2019 in Washington DC (Johns Hopkins Center for Health Security, 2019). Experts in technology, policy, law, and management from China and the United States discussed strategies for dealing with the potential biosafety risks of synthetic biology. The experts at the meeting pointed out that synthetic biology concerns stem from the biosafety risks due to the misuse of synthetic biological techniques by researchers and the potential biosecurity risk of abusing these techniques for terrorism (Johns Hopkins Center for Health Security, 2019). In addition, it is equally important to train and assess the biosafety awareness of researchers working with synthetic biology. The release of experimental products and experimental pathogens, intentional or unintentional, should be identified as a potential biosafety risk, which should be governed under strict regulation. Nevertheless, as a research technology that breaks through the laws of natural evolution, synthetic biology may have many unpredictable potential risks and its sustainable development necessitates a relatively complete and comprehensive governance system. Therefore, a more comprehensive and detailed regulatory system is required to control the development direction of the industry. In addition, although laws and regulations on biosafety and biosecurity concerns have been promulgated by many governments, more efforts are still needed to strengthen the exchange and sharing of knowledge on how to effectively respond to global epidemics like the COVID-19.

## Synthetic Biology, Pandemics, and Its Control Strategies

Synthetic biology is a “double-edged sword.” It can bring more knowledge about microorganisms and diseases for the benefit of humanity. As described in a report on DURC drafted by the National Science Advisory Board for Biosecurity (NSABB), synthetic biology has the potential to raise bio-risk concerns, either intentionally or unintentionally. As mentioned earlier, the concerns of synthetic biology come from the biosafety risks caused by the misuse of synthetic biology techniques by researchers, and potential biosecurity issues resulting from abuse of synthetic biotechnology for bioterrorism or warfare, or by accidental leaks.

The recent outbreak of the novel coronavirus and various speculations about the origin of the virus have attracted more attention on biosafety of synthetic biology and its prevention as well as control of pandemics. Although the source of the virus is not yet known, and there is a lack of convincing proof that the novel coronavirus epidemic is related to biosafety concerns of synthetic biology, the global pandemic caused by the virus has already manifested itself and has caused enormous damage worldwide, including human lives, national economies, and social and moral aspects.

### Synthetic Biology and the Novel Coronavirus SARS-COV-2

The novel coronavirus pandemic has swept across the world in just a few months and has made a quite big impact on the international community. Various hypotheses about the origin of the novel coronavirus SAR-CoV-2, especially the one which states that it was made and leaked out from a laboratory, have aroused great concerns globally. Although scientists have made considerable efforts to explore the origin of SARS-CoV-2, so far it remains an unsolved mystery.

#### The Origin of Novel Coronavirus Remains Hotly Debated and Unresolved

After an outbreak of an epidemic, the search for the origin of the pathogen and the route of transmission has always been of interest to scientists and the public. The origin of the novel coronavirus SARS-CoV-2 is no exception. However, there are no persuasive data on the real onset of SARS-CoV-2 infection and spread in the pre-pandemic period worldwide since there are too many controversies and doubts about the origin of SARS-COV-2.

Based on the genomic analysis of different strains of SARS-CoV-2 (Andersen et al., 2020; Benvenuto et al., 2020; Centers for Disease Control and Prevention, 2020; Lu et al., 2020; Paraskevis et al., 2020; Ren et al., 2020; Wan et al., 2020; Zhou P. et al., 2020; Zhu et al., 2020), 27 scientists, on February 19, 2020, issued a joint statement that the novel coronavirus might originate from wild animals (Calisher et al., 2020). In a review article published on February 26, 2020 in the journal “Emerging Microbes & Infections,” Shan-Lu Liu and others speculated that the novel coronavirus might be a new virus formed by the recombination of bat coronavirus with other viruses in an intermediate host rather than been artificially engineered (Liu et al., 2020), although there lacks direct evidence to supporting their speculation. Internationally renowned viral evolutionists Kristian Andersen and Andrew Rambaut co-published a review article in Nature Medicine entitled “The Proximal Origin of SARS-CoV-2,” indicating that this novel coronavirus is of wildlife origin (Andersen et al., 2020). Nevertheless, the above arguments did little to concerns that SARS-CoV-2 was the result of a laboratory accident or was intentionally engineered (Rasmussen, 2021).

One version of the laboratory origin story relies on the fact that SARS-CoV-2 was engineered for gain-of-function research and has been previously studied with bat SARS-like coronaviruses to understand the risk of cross-species transmission (Menachery et al., 2015; Rasmussen, 2021). Ironically, these gain-of-function research have provided valuable information about the biology of SARS-CoV-2.

From scientific point of view, before we would have obtained solid and convincing evidence to prove its true origin, we could not rule out the following possibilities (1) naturally occurring, (2) unintentionally made and leaked out of the lab, and (3) a combination/extension of (1) and (2), including under-appreciated or unassessed interactions between the man-made and the natural world. Ms. Angela L. Rasmussen recently published a short comment titled “On the origins of SARS-CoV-2” to appeal to the stakeholders in public health—scientists, clinicians and, most importantly, members of the public to understand or study the origins of SARS-CoV-2 using an evidence-driven approach (Rasmussen, 2021). Unfortunately, there is insufficient evidence to prove that it came from natural evolution, neither is there sufficient evidence to prove that it was intentionally made and leaked out of a laboratory. SARS-COV-2 is different enough from the closest published natural strain that it is very unlikely to have been engineered from that strain, but it cannot exclude the possibility that the immediate precursor is an unpublished and unacknowledged natural strain that was in the possession of a laboratory, and we cannot rule out the possibility that some strains could naturally evolved further in an environment of artificially imposed evolutionary pressure or under some unknown extreme natural evolutionary conditions. Compelling evidence of natural origin would be the discovery in nature of the immediate precursors, which has not been done.

There is an extensive history of pathogen emergence by natural routes: most novel viral pathogens that have caused epidemics or pandemics in humans have emerged naturally from wildlife reservoirs. Therefore, prevailing view among many scientists is that this virus could found its way into the human host through a series of unpleasant and unexpected encounters with animals (Rasmussen, 2021) although the true origin of SARS-CoV-2 is still hotly debated and unresolved.

#### The Host of the Novel Coronavirus Remains a Mystery

Some researchers have conducted in-depth research on bat viruses and accumulated a lot of viral genome data. Sequence alignment showed that some viruses carried by bats, such as RaTG13 and RmYN02 have a high degree of similarity with the novel coronavirus, respectively (Zhou H. et al., 2020; Zhou P. et al., 2020). The high correlation between different bat coronaviruses and SARS-CoV-2 suggests that bats are likely hosts for SARS-CoV-2. However, based on the similarity and evolutionary analysis, the differences between SARS-CoV-2 and related bat coronaviruses may represent more than 20 years of natural sequence evolution, suggesting that these bat coronaviruses can be considered as either possible evolutionary precursors of SARS-CoV-2, but not as direct sources of SARS-CoV-2, or the immediate precursors of SARS-COV-2 that could naturally evolve under some unknown extreme evolutionary conditions. Although there are many well-established bacterial or yeast-based gene combination platforms which make it possible to synthesize a living organism, there is lack of convincing evidence to prove or disprove that these platforms could have played some part in the missing intermediate host of SARS-CoV-2.

Since bats and humans have very low possibility of contact chances, one might argue that it is unlikely for bat to be an intermediate host to pass these viruses to humans. An additional argument for a non-bat intermediary is that the spike protein appears to include sequences from not-bat coronaviruses. But even though contact chances between bats and humans are low, they appear to have happened: according to Wang N. et al. (2018), “Serological Evidence of Bat SARS-Related Coronavirus Infection in Humans” 3% of people surveyed in seropositivity study had antibodies to bat coronaviruses. This is not a high ratio but it is certainly not zero. That is, notwithstanding the low contact chances of bats and humans, we still can’t rule out the possibility that bat is an intermediate host.

In addition to bats, pangolin is another wildlife possible host that may be related to SARS-CoV-2 since the receptor binding domain (RBD) on pangolin-borne virus – that part of the virus that binds directly to the receptor by which it gains entry into cells –matches the corresponding part of the SARS-COV-2 virus better than any other virus (Lam et al., 2020; Xiao et al., 2020). Multiple SARS-CoV-2 related viruses were found in the tissues of Malayan pangolin smuggled from Southeast Asia to Southern China from 2017 to 2019. Different research groups isolated and sequenced coronaviruses from the smuggled pangolin intercepted by Guangdong Customs and found 99.8% sequence identity of the viral strains, and their sequence similarity with SARS-CoV-2 was 92.4%. Their RBDs were highly similar to SARS-CoV-2, and only one amino acid difference was found between the receptor binding motifs (RBM) of these viruses and SARS-CoV-2. Unlike healthy bats carrying coronaviruses, coronavirus-infected pangolins showed clinical signs and histopathological changes, including interstitial pneumonia and inflammatory cell infiltration, suggesting that pangolins are unlikely hosts for these coronaviruses and may be infected after spillover from their natural hosts, i.e., they may be intermediate hosts for the virus to humans, but the available data are also insufficient to explain pangolins as intermediate hosts for SARS-CoV-2.

Studies have also reported infection of dogs and cats with SARS-Cov-2, but the possibility of transmission of SARS-CoV-2 from cats to humans is uncertain. For the same reason, we need direct and convincing evidence to confirm the intermediate host relationship between humans and animals.

#### Artificial Synthesis of Novel Coronavirus SARS-Cov-2

On May 4, 2020, scientists from Switzerland, Germany, and Russia reported that they had successfully pieced together synthetic viral gene fragments using the published SARS-CoV-2 sequence. They reconstructed the active novel coronavirus harboring green fluorescent signal in the sequence by using a well-established yeast-based gene combination platform (Thao et al., 2020). By artificially synthesizing the novel coronavirus, it can promote research on viral pathogenic mechanism, drug screening, and vaccine development. However, it also increases the chances of virus leakage or criminals using the information to create more infectious and toxic viruses.

### Synthetic Biology Involved in Pathogen Identification and Vaccine Development

Some countries take COVID-19 as an influenza, but extensive disease characterization data and research have shown that COVID-19 is not comparable to pandemic influenza, either in terms of its health hazards or its potential harm to society (Latreille and Lee, 2021). Evidence indicated that synthetic biology techniques play an important role in developing sensitive and specific diagnostic kits, vaccines and therapeutic drugs during the fighting against COVID-19.

Synthetic biology can facilitate the detection of SARS-CoV-2. The application of CRISPR-Cas technology in pathogen identification has significantly reduced the cost of SARS-CoV-2 detection, with its total cost much lower than that of conventional RT-PCR assay. In scenarios where on-site testing is required, the CRISPR-Cas method offers additional advantages, particularly in terms of time savings (Clyde, 2021; Palaz et al., 2021; Rahimi et al., 2021). Furthermore, the CRISPR-Cas13-based detection technique can distinguish SARS-CoV-2 and its mutants (Wang et al., 2021). Recently, Wang et al. (2020) developed a highly sensitive, much more specific and accurate PfAgo-based detection of SARS-CoV-2, which can also distinguish mutants of coronavirus by combining the programmable nuclease PfAGO with RT-PCR technology (Wang et al., 2020).

With synthetic biology, significant progress has been made in SARS-CoV-2 vaccine development. Currently, most vaccines that have been or are being developed, such as inactivated vaccines, subunit vaccines, viral vector-based vaccines, etc., are based on synthetic biology (Strizova et al., 2021). However, it is of great important to develop effective vaccines under the strict bio-risk assessment and governance (Haynes et al., 2020; Belete, 2021) as serious incidents have been witnessed in the development of vaccines in the past (Haynes et al., 2020). For example, the formalin-inactivated vaccine for Respiratory Syncytial Virus (FI-RSV) was discontinued because of vaccine-associated enhanced disease (VAED). In the current development of the COVID-19 vaccine, serious adverse events have previously triggered the suspension of trials while a comprehensive assessment of causality associated with vaccination was completed by an independent review committee, as was done in the chimpanzee adenovirus vector vaccine study (Phillips et al., 2020; Zimmer et al., 2020).

Biosafety of vaccine permeates the entire vaccine development and use process. In order to be able to implement an effective COVID-19 vaccine as widely, rapidly, and safely as possible, it is necessary to ensure that safety risks (e.g., VAED) are identified, quantified and weighed against potential benefits.

## Conclusion

The rapid development of synthetic biology has led to breakthroughs in biomedical, environmental science, energy, and food industries. However, due to limitations in the understanding of the code of life, as well as the possible intended or unintended uses of the technology, the development and application of synthetic biology has associated with bio-risks that may pose unknown hazards to public health and the environment. In order to manage the issue of bio-risk concerns, there is a great need for legislative and regulatory constraints and oversight when one is working with synthetic biology technologies, especially when dual-use biotechnology is involved. In the event of an outbreak, appropriate measures should be taken for the early prevention and control of epidemic regardless of the origin of the pathogens, and governance of pathogen-related research should be strengthened.

For biosafety and biosecurity concerns, synthetic biology may cause intentional or unintentional risks such as food security, ecological sustainability, despite its enormous economic potential to provide society with more accessible, sustainable, and affordable materials. Thus, the industrialization process of synthetic biology may require some examination and development of existing economic and regulatory agencies. At the same time, the risk of unintended and unpredictable adverse effects on people’s health and the environment should be taken into account when synthesizing organisms, because any negligence and mismanagement in such laboratories involving bio-risk concerns may lead to adverse consequences. In order to avoid such bio-risks pose by synthetic biology, actors working with pathogens should be limited to specially trained professionals, working under strict management and supervision.

Although there are bio-risk concerns with synthetic biology, it remains a powerful tool at our disposal in the face of threats to prevent the spread or recurrence of the same pandemic. Vaccines, pathogen specific antibodies and other drugs should be developed through synthetic biology techniques to reduce the lost of targets in case of pathogen mutations, and appropriated measures that improve the rapid response system for new infectious diseases should be put in place. The development of vaccines and effective drugs using synthetic biology techniques, under the governance of laws and regulations, may be the most practical way to ultimately control the epidemic. As John Glass addressed: “I believe that in my lifetime, we will see someone with nefarious intent use synthetic biology in a bad way to cause mayhem, terrorism, you name it. But I also believe that this same technology is going to save the world. I have faith in what we do and its potential” (Cell., 2018).

## Author Contributions

WA conceived and supervised the study. JL and LZ wrote the initial manuscript, and WA rewrote the manuscript. HZ wrote the section “Biosafety Governance of Synthetic Biology in Different Countries.” WA, HZ, and JL edited, corrected and proofread the full contents of the paper. All authors read and approved the final manuscript.

## Conflict of Interest

The authors declare that the research was conducted in the absence of any commercial or financial relationships that could be construed as a potential conflict of interest.
